# Resolving the Multiple Controls of Biomass Production Efficiency in Woody Plants

**DOI:** 10.1111/gcb.71173

**Published:** 2026-11-04

**Authors:** Ruijie Ding, Rodolfo L. B. Nóbrega, Alessio Collalti, Sandy P. Harrison, Iain Colin Prentice

**Affiliations:** ^1^ Georgina Mace Centre for the Living Planet, Department of Life Sciences Imperial College London Ascot UK; ^2^ Department of Geography and Environmental Science University of Reading Reading UK; ^3^ School of Geographical Sciences University of Bristol Bristol UK; ^4^ Cabot Institute for the Environment University of Bristol Bristol UK; ^5^ Graduate Program in Civil and Environmental Engineering (PPGECA) Federal University of Campina Grande Campina Grande Brazil; ^6^ Forest Modelling Laboratory, Institute for Agriculture and Forestry Systems in the Mediterranean National Research Council of Italy (CNR‐ISAFOM) Perugia Italy; ^7^ Department of Earth System Science, Ministry of Education Key Laboratory for Earth System Modeling, Institute for Global Change Studies Tsinghua University Beijing China

**Keywords:** autotrophic respiration, biomass production efficiency, carbon allocation, mycorrhizal association, soil properties, temperature response, woody plants

## Abstract

Plants assimilate carbon through photosynthesis to support biomass production (BP), autotrophic respiration (*R*_a_) and the synthesis of non‐structural compounds. Biomass production efficiency (BPE), the fraction of assimilated carbon allocated to BP, is one key factor influencing ecosystems' potential to store carbon. However, there is no consensus on the controls of BPE, and even the sign of its response to growth temperature is unclear. By means of statistical hypothesis testing using the largest observational dataset to date on annual BPE (*n* = 514 records) from evergreen and deciduous woody plants, we confirm earlier findings that BPE declines with stand age and is greater in deciduous plants. However, we also show that BPE declines toward more organic, sandy, or alkaline soils and increases with climatic aridity, while declining with warmer growing seasons and increasing with warmer winters. Such a dual temperature response is consistent with additional carbon costs due to the need for cold‐hardening processes, and reconciles contradictory published reports of both positive and negative effects of temperature on BPE. Notably, our analysis provides novel evidence that BPE is significantly higher in species associated with arbuscular mycorrhizal (AM) fungi than in those associated with ectomycorrhizal (EcM) fungi and mixed EcM–AM associations. Our analysis explains the observed BPE patterns better than dynamic global vegetation models (DGVMs) from the TRENDY (Trends and Drivers of the Regional‐Scale Terrestrial Sources and Sinks of Carbon Dioxide) project, which show a weaker correspondence with observations. In particular, our model captures reduced BPE in boreal regions, consistent with the predominance of ectomycorrhizal (EcM) species there. This study advances understanding of the global controls of BPE, and suggests a route toward the improved representation of plant growth in vegetation and land surface models.

## Graphical abstract

Our global synthesis reveals that forest BPE is jointly controlled by seasonal climate, soils, stand development, and biological strategy. Separating growing‐season and winter temperatures reconciles previously conflicting temperature responses, while identifying variation associated with mycorrhizal strategy provides a new biological constraint for global vegetation models.

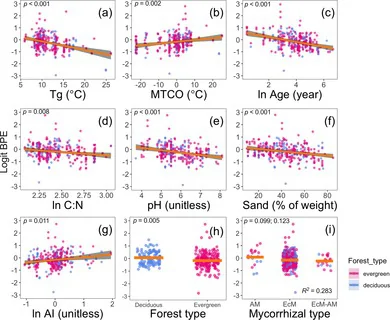

## 1 Introduction

Biomass production (BP) is a central process in the terrestrial carbon cycle, linking photosynthetic carbon uptake by leaves to carbon storage in plants. The amount of carbon that accumulates in plant biomass is determined by the balance of photosynthesis (gross primary production, GPP) and carbon losses, of which the largest is autotrophic respiration (*R*_a_) (Prentice et al. 2024). The fraction of GPP used for BP, known as biomass production efficiency (BPE), expresses the extent to which photosynthetic activity translates into biomass growth.

BPE is related to but not identical with carbon‐use efficiency (CUE), the ratio of net primary production (NPP) to GPP. NPP includes BP as well as the production of non‐structural organic compounds such as storage carbohydrates, root exudates, and volatile organic compounds (VOCs) (Collalti, Ibrom, et al. 2020; Luyssaert et al. 2007). Missing components of NPP have been estimated to account for about 11% of NPP globally and up to 20% of the sum of foliage, wood, and root production in tropical forests (Luyssaert et al. 2007). Biomass pools within BP also differ in turnover time, from rapidly replaced leaves and fine roots to long‐lived woody tissues. However, these additional fluxes are usually difficult to quantify at large spatial scales, and most of the relevant studies have not distinguished NPP from BP (Malhi et al. 2009, 2011; Collalti, Ibrom, et al. 2020). We therefore analyze BPE and CUE together, following the approach of Collalti, Ibrom, et al. (2020), while recognizing the resulting imprecision.

Early experimental studies contributed to the view that plant growth efficiency was relatively invariant across environmental conditions. Waring et al. (1998) suggested a global mean BPE of 0.47 ± 0.04 (mean ± standard deviation; *n* = 12) across temperate and boreal forests, while Van Oijen et al. (2010) proposed that physiological and mass‐balance constraints restrict BPE to a relatively narrow range of 0.5–0.6. A single universal BPE value has been assumed in several vegetation and forest models, including CASA (Potter et al. 1993), FOREST‐BGC (Running and Coughlan 1988), 4C (Lasch et al. 2005), BASFOR (Van Oijen et al. 2005), 3‐PG (Landsberg and Waring 1997), and early versions of 3D‐CMCC FEM (Collalti et al. 2014), which simplifies carbon‐cycle modeling.

However, constant BPE is incompatible with the alternative simplifying assumption adopted in many vegetation and land surface models, in which photosynthesis and respiration are to some extent decoupled. In these models, *R*_a_ is determined independently by biomass and the short‐term temperature response of enzyme kinetics, rather than being proportional to GPP (Collalti, Tjoelker, et al. 2020). This last point has sparked discussion, as rising temperatures would then be expected to increase *R*_a_ while their effects on GPP could be positive or negative (Drake et al. 2016). If GPP declines with increasing temperature, or if the increase in *R*_a_ exceeds that of GPP, warming would reduce BPE (Drake et al. 2016), with negative values in extreme cases.

Empirical analyzes challenge *both* the assumption of constant BPE *and* the hypothesis that BPE declines with temperature due to increasing *R*_a_ (Collalti, Ibrom, et al. 2020; Niu et al. 2024). Available evidence instead suggests that *R*_a_ is controlled by both environmental conditions and biotic factors such as forest type and age, while its climatic response differs from the steep short‐term enzymatic response to temperature (Collalti, Ibrom, et al. 2020). A clear picture of these controls has not yet emerged. BPE has been reported either to remain relatively unchanged (Doughty et al. 2018) or to increase with mean annual air temperature (Piao et al. 2010; Collalti, Ibrom, et al. 2020). BPE has also been reported to increase with absolute latitude (Collalti, Ibrom, et al. 2020), while its relationship with precipitation varies among studies (Collalti, Ibrom, et al. 2020; Luo et al. 2025; Malhi et al. 2015; Migliavacca et al. 2021). Regional and global studies have also reported a positive effect of soil fertility on BPE (De Lucia et al. 2007; Vicca et al. 2012; Doughty et al. 2018).

In contrast, BPE generally declines with forest age (Collalti, Ibrom, et al. 2020), potentially reflecting the increasing mass of respiring sapwood required to support a given leaf area as trees grow taller (Collalti, Tjoelker, et al. 2020; Lasch et al. 2005; Mäkelä and Valentine 2001). This process may also underlie the strong effect of management on BPE shown by Campioli et al. (2015). Age‐related declines in forest growth may also reflect reduced GPP under increasing hydraulic limitation (Hubbard et al. 1999; Ryan and Yoder 1997; Ryan et al. 1997) as well as the effects of increasing *R*_a_ with tree height. BPE has been found to be greatest in temperate deciduous forests and lowest in boreal forests (De Lucia et al. 2007; He et al. 2020; Lasch et al. 2005; Luyssaert et al. 2007; Vicca et al. 2012).

Vegetation effects on ecosystem functioning are mediated by mycorrhizae, the plant–fungal associations formed by most plant species. Ecosystems dominated by ectomycorrhizal (EcM) and ericoid mycorrhizal (ErM) plants often show higher soil carbon‐to‐nitrogen (C:N) ratios than those dominated by arbuscular mycorrhizal (AM) plants, reflecting lower nutrient availability that may constrain BPE (Averill et al. 2014; Phillips et al. 2013; Read et al. 2004; Terrer et al. 2019). The assumption of constant BPE therefore fails to capture non‐negligible variation among forest types, mycorrhizas, stand ages and management regimes (Averill et al. 2014; Campioli et al. 2015; Collalti, Ibrom, et al. 2020; De Lucia et al. 2007; Luyssaert et al. 2007; Mäkelä and Valentine 2001; Phillips et al. 2013; Read et al. 2004; Terrer et al. 2019; Vicca et al. 2012), as well as environmental influences.

Given the uncertainty about the controls of BPE, it is unsurprising that models diverge in their representations of BPE (Bloom et al. 2016; Wei et al. 2022). The temperature sensitivity of BPE, in particular, differs among models. Moderate Resolution Imaging Spectroradiometer (MODIS) derived estimates of BPE show a steeper decline in BPE with increasing temperature, particularly in warm and dry regions, than five dynamic global vegetation models (DGVMs) (He et al. 2018). This difference may reflect the high temperature sensitivity assigned to *R*_a_ in the MODIS NPP product, which assumes no temperature acclimation (Ise et al. 2010). The decline in BPE with increasing temperature is buffered by respiratory acclimation, which adjusts the base rate of respiration to growth temperature and weakens the response of *R*_a_ to sustained warming relative to its short‐term kinetic response (Atkin et al. 2015; Drake et al. 2016; Ren et al. 2024; Wang, Atkin, et al. 2020; Zhang et al. 2025). However, DGVMs consistently show declining BPE with temperature (even when they allow some degree of respiratory acclimation), conflicting with the increase inferred from data by Collalti, Ibrom, et al. (2020).

In this study, we re‐assess the determinants of forest BPE. Our analysis is based on the largest observation‐based global dataset to date (*n* = 514 records), including forest CUE and BPE measurements in evergreen and deciduous woody species, across ecosystems ranging from the tropics to the high latitudes (42.52° S to 64.20° N). We adopted a hypothesis testing approach, selecting seven variables as quantitative predictors in a multiple regression based on their hypothesized relevance to BPE: mean *growing‐season* air temperature (*T*_g_); mean air temperature of the coldest month (MTCO); stand age; soil C:N ratio, soil pH, sand content; and a climatic aridity index (AI). Distinguishing growing‐season temperature from winter temperature, rather than relying on mean *annual* temperature, we tested whether growing‐season metabolism and cold‐season maintenance costs exert contrasting controls on BPE and quantified the relationships between BPE and edaphic and biological factors. We tested the following seven hypotheses:

- BPE *decreases* with *T*_g_, due to *R*_a_ increasing faster with growth temperature than photosynthesis (Zhang et al. 2014).
- BPE *increases* with MTCO, because the extra respiratory costs related to entering, maintaining, and breaking winter dormancy in cold climates are reduced or absent in warmer climates (Chang et al. 2021; Sperling et al. 2015).
- Taller trees respire more (relative to GPP) because of their greater volume of living sapwood per unit leaf area: leading to increased *R*_a_, and thus reduced BPE (Collalti et al. 2019; West 2020).
- Plants on nitrogen‐poor (high C:N ratio) soils allocate more biomass below ground for nutrient acquisition, leaving less for allocation to leaves; this lowers BPE (Doughty et al. 2018; Ding et al. 2025; Vicca et al. 2012).
- Plants on more acid (lower pH) soils allocate less biomass below ground for resource acquisition, leading to higher BPE (Ding et al. 2025; Wang et al. 2024).
- Sandy soils retain less water and nutrients. Water stress and/or nutrient limitation require greater below‐ground carbon allocation, leading to reduced BPE (Ding et al. 2025; Jiménez et al. 2014).
- Greater climatic aridity increases BPE by reducing respiratory and turnover losses relative to carbon assimilation through cost‐minimizing strategies, even as GPP declines (Ding et al. 2025; Luo et al. 2025; Malhi et al. 2015).

## 2 Methods

### 2.1 BPE and Climate Data Compilation

The data were assembled from three literature sources containing compilations of CUE or BPE (Collalti, Ibrom, et al. 2020; Peng et al. 2023; Xu et al. 2014). These sources include geographic coordinates, vegetation (biome and dominant species), GPP, NPP, BP, forest type and age. Reported NPP is considered as BP because non‐structural and secondary carbon compounds (e.g., BVOCs or exudates) are not included in most published estimates of NPP. Globally, 319 records are from temperate sites (23.5°–50°), 133 from boreal sites (> 50°), and 78 from tropical sites (0°–23.5°), corresponding to 166 deciduous, 348 evergreen, and 16 mixed forests. However, samples from mixed forests were not included in the analysis.

The climate variables (temperature; vapor pressure deficit, VPD; precipitation; solar radiation) were extracted from CHELSA V2.1, with a horizontal resolution of 30 arc sec, monthly from 1982 to 2019 (Karger et al. 2017). Monthly temperature values were averaged over the thermal growing season, defined as the period with climatological daily mean temperatures > 0°C, to obtain *T*_g_. MTCO is the mean temperature of the coldest month. Photosynthetic photon flux density (PPFD) was derived from shortwave downwelling radiation. The aridity index (AI) is the ratio of potential evapotranspiration to annual precipitation, where potential evapotranspiration was estimated using version 2 of the Simple Process‐led Algorithms for Simulating Habitats (SPLASH) model (Sandoval et al. 2024). Soil pH data were derived from the gridded Global Soil Dataset (GSDE) with 30 arc sec resolution (Wei et al. 2014). Soil sand and clay content and bulk density were derived from SoilGrid2.0 with 250 m resolution (Poggio et al. 2021), and soil C:N from WISE30sec data with a resolution of 30 arc sec (Batjes 2015). Soil plant‐available phosphorus (P) was obtained from the Global Database of Soil Plant Available Phosphorus at a resolution of 1 km^2^ (McDowell et al. 2023).

Direct records of forest management were not available for the BPE observation sites; therefore, forest management information was obtained from the Global forest management data for 2015 at 100 m resolution (Lesiv et al. 2018). This dataset classifies forests into six management categories: naturally regenerating forests without signs of management, including primary forests; naturally regenerating forests with signs of management (e.g., logging or clear‐cutting); planted forests; plantation forests with rotation times up to 15 years; oil palm plantations; and agroforestry systems. Species‐level mycorrhizal association data were obtained from the “FungalRoot” database, a global compilation of plant mycorrhizal associations (Soudzilovskaia et al. 2020). In this compilation, spatial patterns of mycorrhizal vegetation were derived from the global mycorrhizal plant distribution map, based on multiple published datasets, using a number of conversion factors to obtain per pixel values of mycorrhizal plants biomass fractions (Soudzilovskaia et al. 2019). Mixed ectomycorrhizal and arbuscular mycorrhizal colonization (EcM–AM) is included in the “FungalRoot” database at the species level but not explicitly represented in the global mycorrhizal plant distribution map; therefore, an ecosystem‐level EcM–AM category was derived following the approach of Soudzilovskaia et al. (2019). A mycorrhizal strategy was considered dominant when it accounted for more than 20% of total vegetation biomass within a grid cell; grid cells were classified as EcM–AM when both ectomycorrhizal (EcM) and arbuscular mycorrhizal (AM) biomass fractions exceeded this threshold, representing ecosystem‐level co‐dominance rather than a distinct mycorrhizal association.

### 2.2 Empirical Model

A total of 15 predictors were considered in this study, including climate variables (*T*_g_, MTCO, PPFD, VPD and AI), soil properties (C:N ratio, pH, sand content, clay content, bulk density, and plant‐available P), stand age, forest type, mycorrhizal type and forest management. To reduce redundancy among predictors and identify the most influential variables, we applied both bidirectional stepwise regression and LASSO (Least Absolute Shrinkage and Selection Operator) regression (Tibshirani 1996). Stepwise regression was used to explore a broad set of potentially important predictors. LASSO regression applies a penalty term that shrinks coefficients of less important variables toward zero. Variables with strongly skewed distributions, including PPFD, VPD, AI, stand age, soil C:N ratio, and plant‐available P, were natural log‐transformed prior to both analyzes (Figure S1). Coefficients and statistical significance of environmental variables from both stepwise and LASSO regressions are reported in Table S1. Results from the two selection methods were broadly consistent and identified *T*_g_, MTCO, stand age, AI, and three soil properties (C:N ratio, sand content, and pH), as well as forest type and mycorrhizal type, as the strongest predictors of BPE. In contrast, clay content, bulk density, PPFD, VPD, plant‐available P, and forest management were not selected, or were not significant, in either regression approach.

Based on these results, we fitted a parsimonious model using ordinary least squares (OLS) multiple linear regression using the logit‐transform of BPE (which ensures that the transformed variable is unbounded both above and below). The fitted model included nine abiotic and biotic predictors (*T*_g_, MTCO, stand age, soil C:N, pH, sand content and AI), as well as fixed effects for forest type and mycorrhizal type:

(1) $$\begin{array}{cc} \text{logitBPE}\;= & \;\beta_{0}+\beta_{1}T_{g}+\beta_{2}\text{MTCO}+\beta_{3}\text{lnAge}+\beta_{4}\mathrm{lnC}:N\\ & +\beta_{5}\mathrm{pH}+\beta_{6}\text{Sand}+\beta_{7}\mathrm{AI}+\text{Forest type}\\ & +\text{Mycorrhizal type}+\varepsilon \end{array}$$

where *β*_0_ is the intercept, *β*_1_–*β*_7_ are the estimated sensitivities of BPE to *T*_g_, MTCO, stand age, soil C:N, pH, sand content and AI; “Forest type” is a fixed effect for evergreen and deciduous classes; “Mycorrhizal type” includes arbuscular mycorrhizal (AM), ectomycorrhizal (EcM), and mixed EcM–AM associations; and ε is the residual term (Table 1). Model assumptions were evaluated using standard residual diagnostics, but showed no strong violations of linear model assumptions.

**TABLE 1:** Summary statistics for the regression of logit‐transformed biomass production efficiency (BPE) on predictor variables in evergreen and deciduous species.

Predictor variable	Coefficient	SE	*t*‐value	*p*	*R* ^2^	df
Intercept	4.539	0.670	6.779	< 0.001		
*T*_g_ (°C)	−0.071	0.012	−5.708	< 0.001		
MTCO (°C)	0.015	0.005	3.147	0.002		
ln Age (year)	−0.204	0.035	−5.826	< 0.001		
ln C:N	−0.534	0.198	−2.686	0.008		
Sand (% of weight)	−0.009	0.002	−4.252	< 0.001	0.283	319
pH (unitless)	−0.193	0.047	−4.126	< 0.001		
ln AI	0.211	0.083	2.558	0.011		
Forest type: evergreen	−0.226	0.081	−2.796	0.005		
Mycorrhizal_type:EcM	−0.236	0.143	−1.654	0.099		
Mycorrhizal_type:EcM‐AM	−0.310	0.200	−1.548	0.123		

*Note:* Predictor variables are growing‐season mean temperature (*T*_g_, °C), mean temperature of the coldest month (MTCO, °C), natural log‐transformed stand age (ln Age, year), natural log‐transformed soil C:N ratio (ln C:N, unitless), soil pH (pH, unitless), and soil sand content (Sand, % of weight), and natural log‐transformed aridity index (ln AI, unitless). Forest type is represented by (evergreen; deciduous as the reference level), while mycorrhizal type is represented by EcM (ectomycorrhizal) and EcM‐AM (mixed ectomycorrhizal and arbuscular mycorrhizal colonization), with AM (arbuscular mycorrhizal) as the reference level.

We evaluated model performance using 100 repetitions of site‐grouped five‐fold cross‐validation. Sites were defined as unique combinations of latitude and longitude, and all observations from the same site were assigned to the same fold (Roberts et al. 2017). In each repetition, approximately 80% of sites were used for model fitting and 20% for validation. Performance was evaluated using the cross‐validated coefficient of determination (*R* ^2^), root mean squared error (RMSE) and mean absolute error (MAE) on the logit‐transformed BPE scale (Table S2; Figure S2). To assess potential differences in responses between forest types, we also fitted models for evergreen and deciduous plants separately, allowing the responses of logit BPE to all predictors to vary between the two groups (Tables S3 and S4). Although predictor effects differed in magnitude and significance between evergreen and deciduous species (Tables S3 and S4), the direction of the main effects was broadly consistent between evergreen and deciduous groups overall and we therefore present results from the combined model here. We also fitted a linear mixed‐effects model with site included as a random intercept, and the resulting effect directions and relative magnitudes were consistent with those of the OLS model (Table S5).

We applied the LASSO technique using the glmnet R package (Tay et al. 2023). We used the R function lm from the R‐package stats to fit multiple linear models to the data (R Core Team 2024). Regression relationships were visualized using partial residual plots generated by the visreg package in R (Breheny and Burchett 2017). Partial residual plots display the relationship between the response variable and each predictor variable in turn, using the fitted model to set all other predictors constant at their median values. We checked for potential problems of multicollinearity using the variance inflation factor (VIF) calculated in the car package (Fox and Weisberg 2019). All calculations and analyzes were performed using the open‐source environments R version 4.4.1 (R Core Team 2024) and Python version 3.12.

### 2.3 Global Mapping and Vegetation Models

The empirical models—developed based on site‐specific observations—were applied globally, driven by gridded data. We used the gridded climate, soil texture and mycorrhizal data discussed above, but stand‐age gridded data were taken from GFADv1.0 on a 0.5‐degree grid, calculated as the mean age across four plant functional types (PFTs) and 15 age classes (from 0–10 to 140–150 years), weighted by their respective fractional area coverage within each grid cell (Poulter et al. 2018). Global maps were initially created for evergreen and deciduous species separately (see Equation 1). To produce the final BPE values, we compared the dominant species group within each grid cell while also accounting for the dominant mycorrhizal strategy. The species distribution map was developed by compiling a comprehensive global dataset for 16 functional types (FTs) and characterizing trait–trait and trait–climate relationships separately for woody deciduous and woody evergreen groups, using multivariate analysis and generalized additive models (GAMs) (Li and Prentice 2024; Puchi et al. 2026).

We compared our global simulations of BPE with eight dynamic global vegetation models (DGVMs) from version 8 of the TRENDY model ensemble (Sitch et al. 2024), driven by varying CO_2_ and climate and fixed land use (the S2 simulation protocol) (Table 2) (Li and Prentice 2024). BPE in TRENDY was estimated as NPP/GPP for the forest PFTs simulated in each grid cell. NPP is obtained in these models simply by subtracting *R*_a_ from GPP, since the Note that TRENDY models either partially or entirely neglect non‐structural carbohydrates, volatile organic compounds, and root exudates—the components that differentiate NPP from BP, and thus CUE from BPE.

**TABLE 2:** Summary of the process‐based models used with associated product resolution.

Model name	Spatial resolution	Soil resources	References
CABLE	1° × 1°	Soil moisture, temperature	Haverd et al. (2018)
CLASSIC	2.8125° × 2.8125°	Soil moisture, temperature, net radiation, soil texture	Seiler et al. (2021)
ISAM	0.5° × 0.5°	Soil nitrogen	Meiyappan et al. (2015)
ISBA‐CTRIP	1° × 1°	Soil moisture, temperature, soil texture	Decharme et al. (2019)
JSBACH	1.875° × 1.875°	Soil hydrology	Mauritsen et al. (2019)
LPJ‐GUESS	0.5° **×** 0.5°	Soil temperature, moisture, soil texture and soil nitrogen	Smith et al. (2014)
ORCHIDEE	0.5° **×** 0.5°	Soil water, temperature, nitrogen, phosphorus	Goll et al. (2017)
SDGVM	0.5° **×** 0.5°	Soil nitrogen, phosphorus	Walker et al. (2017)

We generated global gridded BP estimates by multiplying BPE values derived from our regression model by gridded GPP simulated using the P model (Stocker et al. 2020), with soil moisture limitation represented by a simple empirical correction factor. To assess the sensitivity of BP estimates to the choice of GPP product, we repeated this calculation using ensemble‐mean GPP from eight TRENDY models while retaining the same empirical BPE estimates and spatial mask. We compared the resulting BP estimates using area‐weighted global totals, pixel‐wise spatial correlation and normalized differences. The theory underlying the P model has been described by Prentice et al. (2014) and Wang et al. (2017). The model combines an optimality‐based theory for the prediction of light use efficiency (LUE) with data on incident photosynthetically active radiation (PAR) and the fraction of absorbed PAR (fAPAR). As inputs for the P model we used *T*_g_, VPD, PAR, fAPAR, and mean atmospheric pressure calculated by the barometric formula using elevation data extracted from the GTOPO30 digital elevation model. VPD and short‐wave radiation (SW) data, used to calculate PAR, were also from CHELSA V2.1, with a horizontal resolution of 30 arc sec, monthly from 1982 to 2019 (Karger et al. 2017). Fluxes per unit leaf area to fluxes per unit ground area (fAPAR) data from 1982 to 2019 at 0.05° spatial resolution (Jiang and Ryu 2016) were derived from the MODIS MOD15A2H LAI/FPAR product (Myneni et al. 2015).

## 3 Results

CUE and BPE were statistically indistinguishable in our dataset, and we therefore considered both metrics together in our analyzes. The average BPE was 0.44 ± 0.14 (range 0.05–0.94; *n* = 514). Mean BPE was 0.48 ± 0.13 for deciduous trees (range: 0.23–0.79; *n* = 166) and 0.41 ± 0.14 for evergreen trees (range: 0.05–0.94; *n* = 348) respectively (Figure 1). Both evergreen and deciduous forests occurred across wide ranges of climate and soil conditions (Figure S1).

**FIGURE 1:**
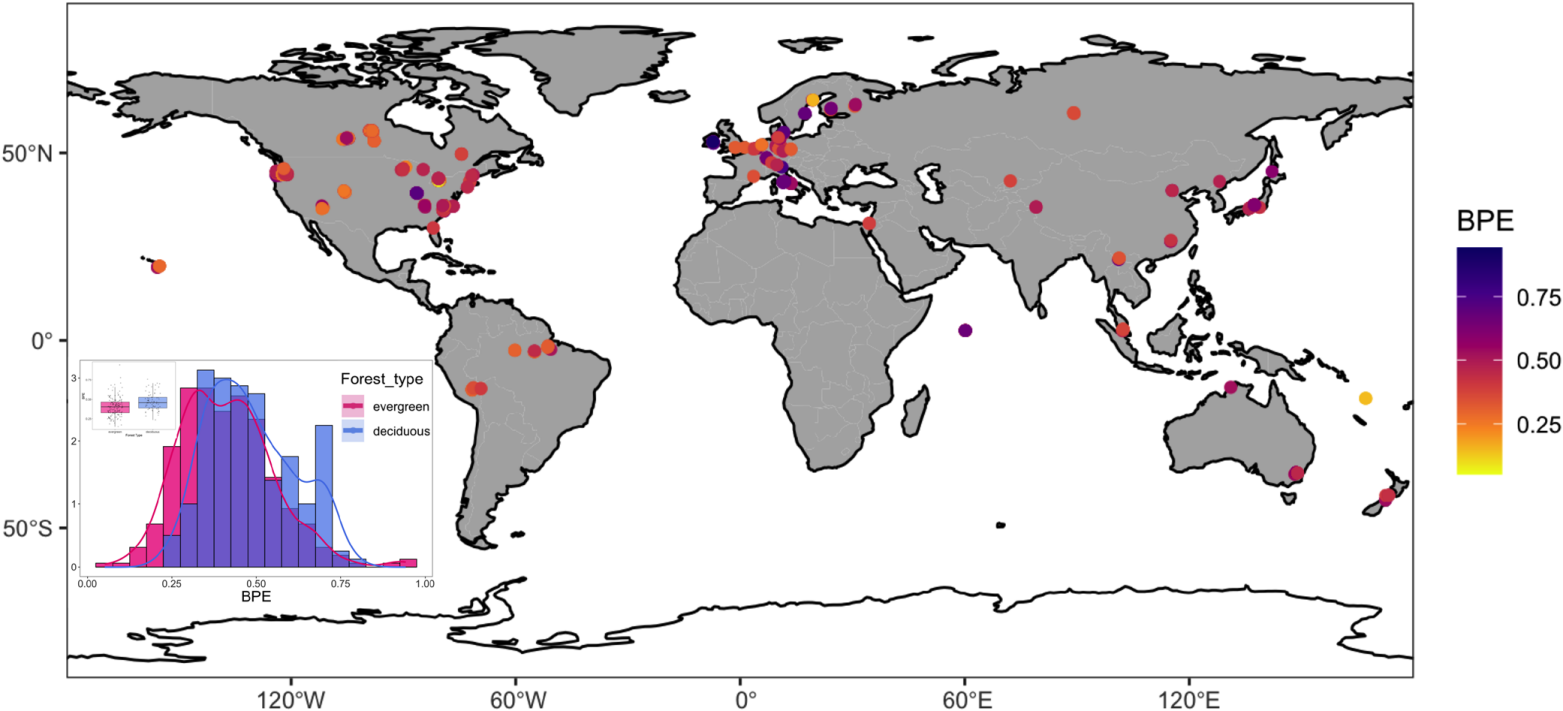
Global biomass production efficiency (BPE) sample locations and density. The database contains 514 BPE records (348 evergreen plants, 166 deciduous plants). The inset shows the distributions of BPE values within the two classes.

Multiple linear regression showed that BPE decreased with *T*_g_ (*p* < 0.001), stand age (*p* < 0.001), soil C:N (*p* = 0.008), pH (*p* < 0.001) and sand content (*p* < 0.001), but increased with AI (*p* = 0.011) and MTCO (*p* = 0.002) (Figure 2a–g; Table 1). In the multiple regression model, deciduous woody plants had higher BPE than evergreen plants (*p* = 0.005). The baseline BPE in deciduous plants was 5% higher than in evergreen plants (Figure 2h). The coefficients for EcM and mixed EcM–AM associations indicated lower BPE relative to AM associations, although these effects were only marginally significant in the combined model (*p* = 0.099; 0.123; Figure 2i). The model explained 28.3% of the variation in BPE (*R* ^2^ = 0.28). Repeated site‐grouped five‐fold cross‐validation showed that the model explained an average of 16.3% of the variation across sites (*R* ^2^ = 0.16), with an RMSE of 0.610 and MAE of 0.461, indicating lower but consistently positive predictive performance (Table S2; Figure S2). Stand age had the highest relative importance, followed by forest type and soil C:N ratio (Figure 3).

**FIGURE 2:**
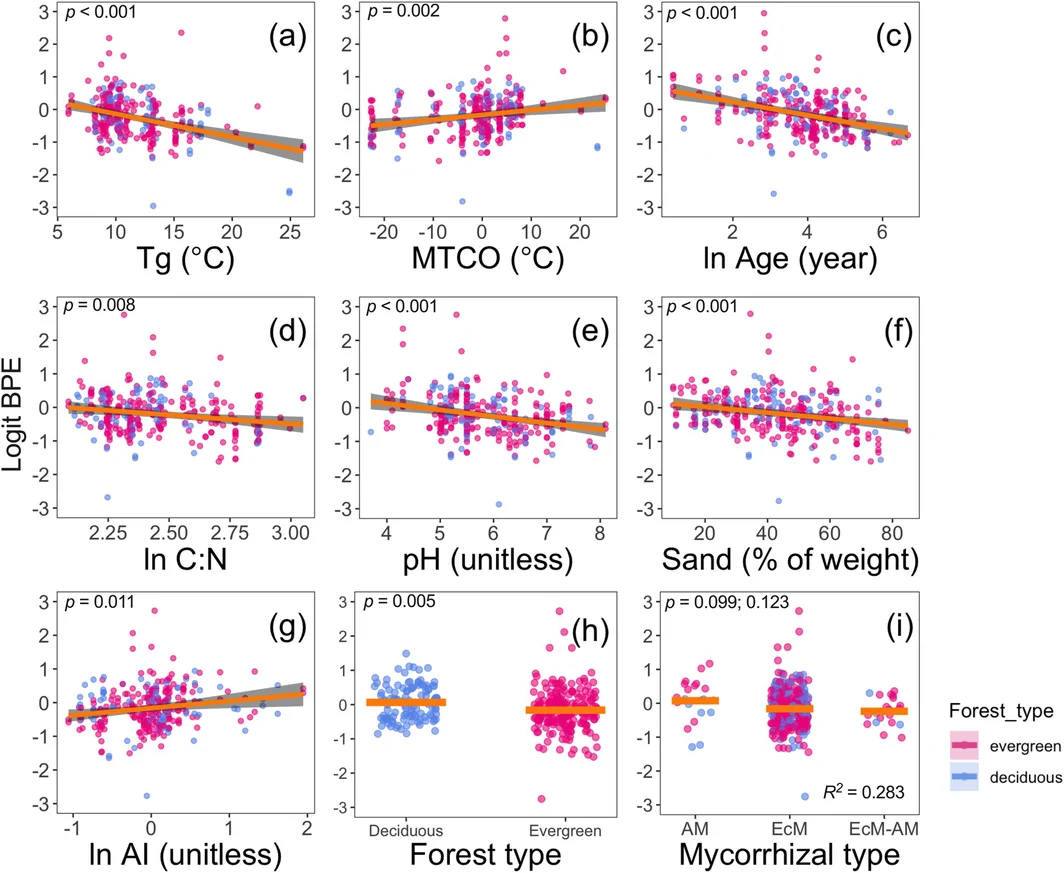
Partial residual plots from the regression of logit‐transformed biomass production efficiency (BPE) against explanatory variables. The plots are from the ordinary least‐squares multiple linear regression in Table 1. (a) Logit ‐transformed BPE response to growing‐season mean temperature (*T*_g_, °C). (b) Mean temperature of the coldest month (MTCO, °C). (c) Natural log‐transformed stand age (ln Age, year). (d) Natural log‐transformed soil C:N ratio (ln C:N, unitless). (e) Soil pH (pH, unitless). (f) Soil sand content (Sand, % of weight). (g) Natural log‐transformed aridity index (ln AI, unitless). (h) Deciduous (blue) versus evergreen (pink). (i) Mycorrhizal type (AM, arbuscular mycorrhizal; EcM, ectomycorrhizal; EcM‐AM, mixed ectomycorrhizal and arbuscular mycorrhizal colonization). Coefficients and standard errors for the fitted lines are given in Table 1.

**FIGURE 3:**
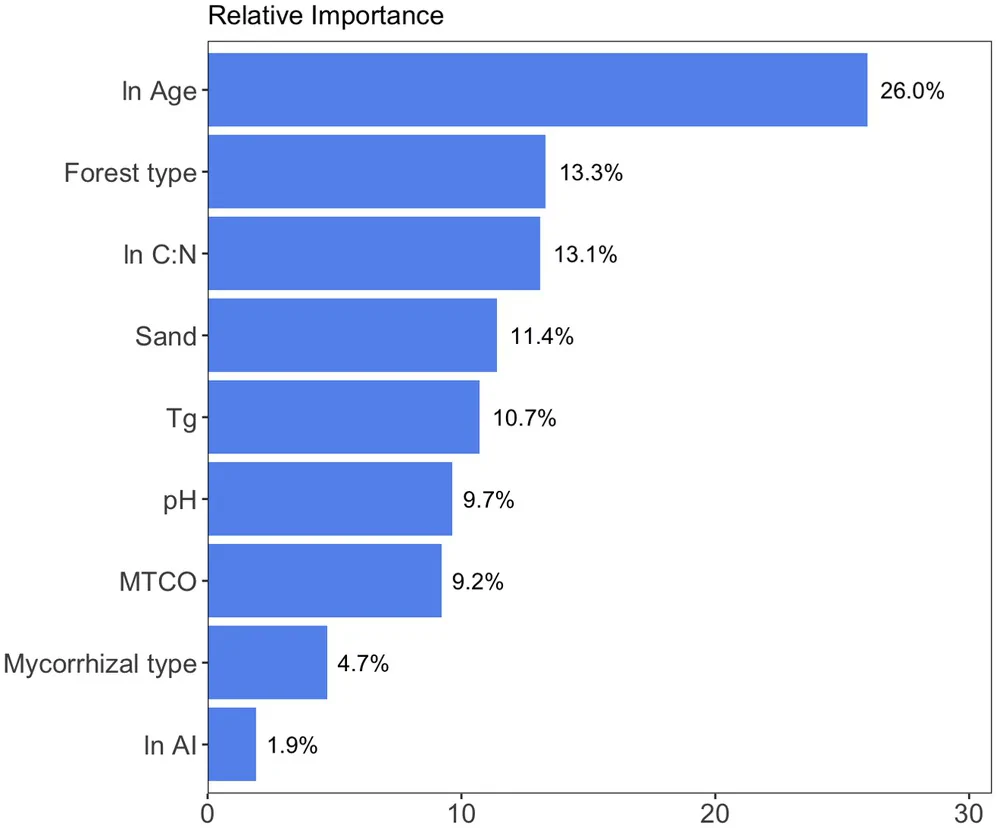
Importance of explanatory variables in the regression model. Numbers represent relative importance as indicated by the Lindeman–Merenda–Gold (LMG) statistic. Explanatory variables are growing‐season mean temperature (*T*_g_, °C), mean temperature of the coldest month (MTCO, °C), natural log‐transformed stand age (ln Age, year), natural log‐transformed soil C:N ratio (ln C:N), soil pH (pH, unitless), soil sand content (Sand, % of weight), natural log‐transformed aridity index (ln AI, unitless), forest type and mycorrhizal type.

The evergreen and deciduous models were also fitted independently but showed broadly similar patterns. In both forest types, *T*_g_, stand age, soil C:N ratio and sand content negatively affected BPE, while AI and MTCO positively affected BPE. BPE in evergreen plants was also negatively associated with soil pH, while this relationship was non‐significant among deciduous plants (Tables S3 and S4). The evergreen model explained slightly less variation (*R* ^2^ = 0.31) than the deciduous model (*R* ^2^ = 0.38) (Tables S3 and S4). While all predictors except mycorrhizal type were significant in the evergreen model, with similar coefficient values to the combined model, only stand age, soil C:N ratio and the EcM and the EcM–AM categories were statistically significant in the deciduous model (with *p* = 0.1 for *T*_g_). The mixed‐effects model produced results consistent with the OLS model after accounting for site‐level variation (Table S5). Simple correlations between BPE and predictors ranged from −0.40 to 0.55 (Figure S3), supporting the use of multiple regression to distinguish their respective effects (Collalti, Ibrom, et al. 2020). The variance inflation factors (VIFs) for the predictors were low, ranging from 1.08 to 1.38 (Figure S3), indicating that multicollinearity is not a problem.

We also assessed the individual contributions of the same predictors used in the BPE model to the data on GPP and BP (Figure 4). The hierarchy of contributions to GPP and to BP is not the same. EcM, soil sand content, soil C:N and *T*_g_ all show significant negative effects on BP but not GPP (Figure 4). Both GPP and BP showed significant negative effects of soil pH and positive effects of MTCO.

**FIGURE 4:**
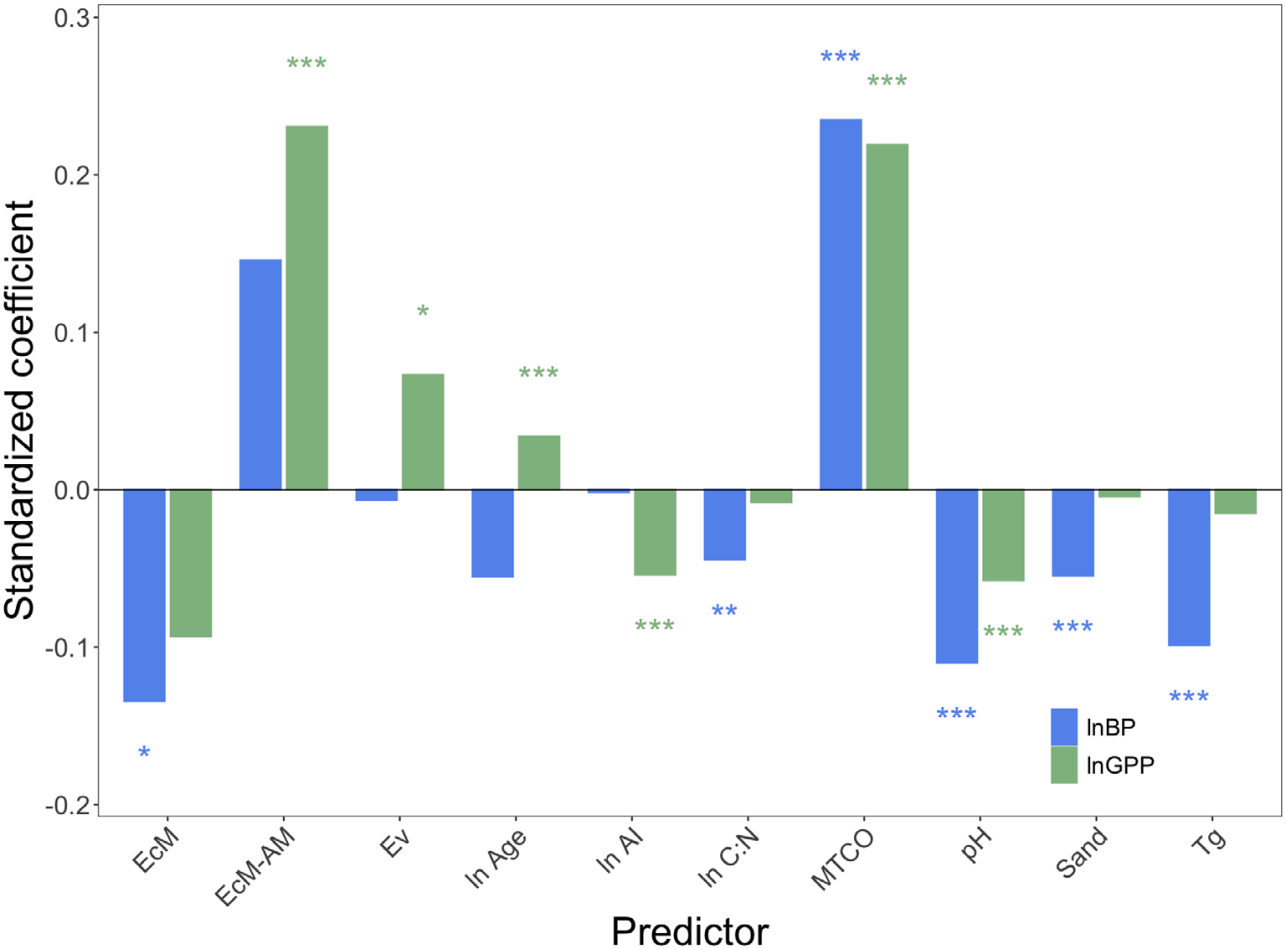
Comparison of the standardized coefficients for the predictors in the models for log‐transformed biomass production (BP) and gross primary production (GPP). Bars represent the standardized coefficients of the predictors, with positive or negative values indicating the direction of the relationship. Continuous predictors were standardized (mean = 0, SD = 1) to obtain standardized coefficients. The color of the bars corresponds to the response variable (ln BP in blue and ln GPP in green). Significance levels are indicated by stars (***p < 0.001, **p < 0.01, *p < 0.05). Predictor variables are growing‐season mean temperature (*T*_g_, °C), mean temperature of the coldest month (MTCO, °C), natural log‐transformed stand age (ln Age, year), natural log‐transformed soil C:N ratio (ln C:N), soil pH (pH, unitless), soil sand content (Sand, % of weight) and natural log‐transformed aridity index (ln AI, unitless). Forest type is represented by Ev (evergreen; deciduous as the reference level), while mycorrhizal type is represented by EcM (ectomycorrhizal) and EcM‐AM (mixed ectomycorrhizal and arbuscular mycorrhizal colonization), with AM (arbuscular mycorrhizal) as the reference level.

**FIGURE 5:**
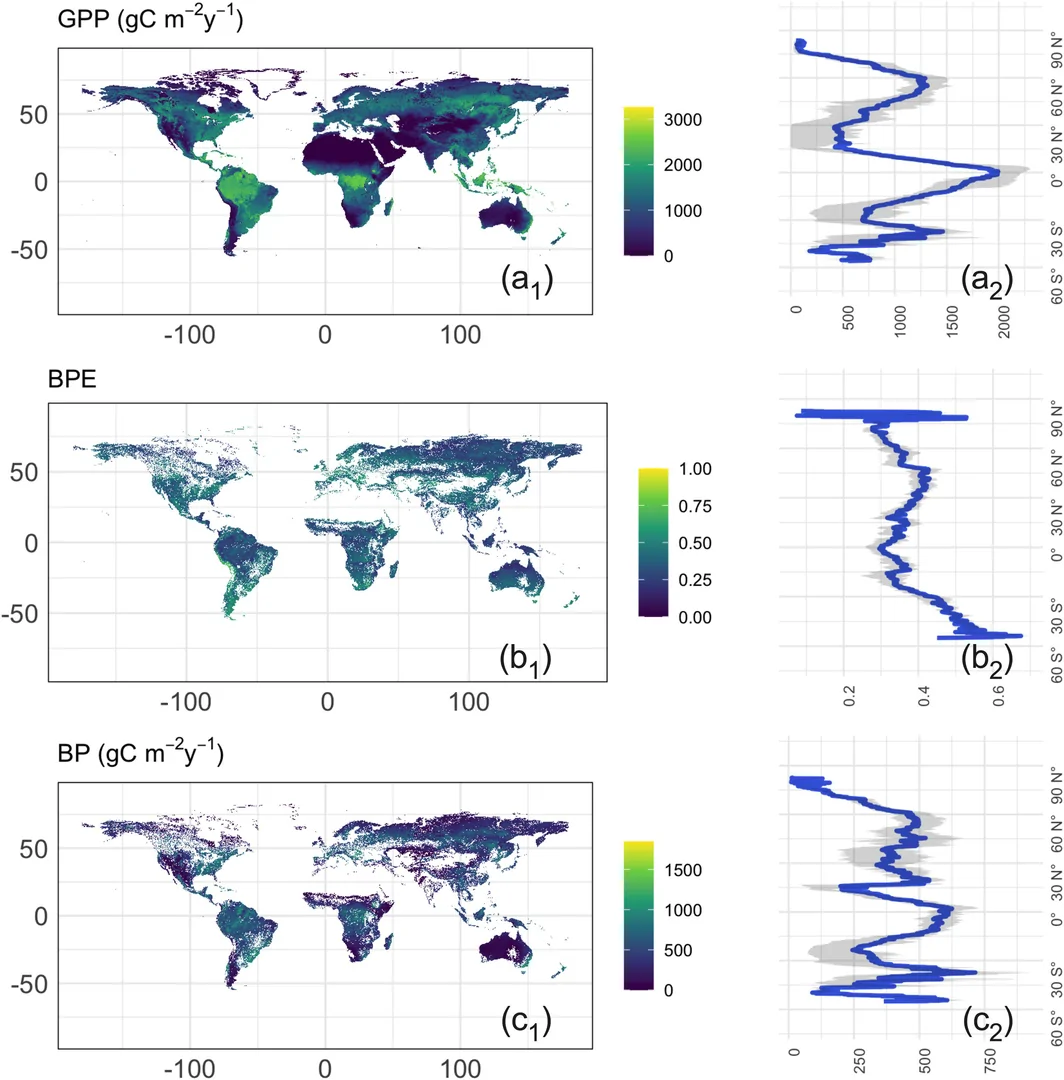
The global spatial distribution of modeled Gross Primary Production (GPP), Biomass Production Efficiency (BPE), and Biomass Production (BP). (a_1_) GPP (gC m^−2^ d^−1^) as predicted by the P model. (b_1_) BPE as predicted by the multiple linear regression model. (c_1_) BP (gC m^−2^ d^−1^), the product of GPP and BPE. (a_2_–c_2_) show latitudinal variations (with standard deviation in grey).

GPP was independently simulated using the P model (Stocker et al. 2020), yielding a maximum value of = 3300 g C m^−2^ yr.^−1^ (Figure 5a_1_,a_2_). A global map of BPE was generated using the multiple regression model, with predicted values ranging from 0.05 to 0.76. The estimates showed a clear latitudinal gradient, increasing from the tropics toward high latitudes (Figure 5b_1_,b_2_). BP was calculated as the product of modeled GPP and BPE. The resulting modeled BP values reached a maximum of = 1847 g C m^−2^ yr.^−1^, with the highest values in regions where GPP is also high (Figure 5c_1_,c_2_). The sensitivity analysis based on the TRENDY ensemble‐mean GPP yielded a total BP of 28.85 Pg C yr.^−1^ across the common mapped forest domain, compared with 33.99 Pg C yr.^−1^ based on P‐model GPP. The two estimates were moderately correlated spatially (*r* = 0.71), indicating somewhat consistent broad‐scale patterns but sensitivity in their magnitude and regional distribution (Figure S4).

Comparing our regression‐based BPE estimates with the measurements from the complete observational dataset yielded an *R* ^2^ = 0.30, with a slope of 0.93 for the empirical model (Figure S5). The TRENDY models showed generally poor agreement with the observed BPE values, with lower, often near‐zero *R* ^2^ values and regression slopes ranging from −0.27 to 0.73. The performance of the ORCHIDEE model was closest to that of our benchmark model, with an *R* ^2^ of 0.14 and a regression slope of 0.73 (Figure S5). BPE estimates from the TRENDY models generally showed a similar latitudinal pattern to the data, increasing from the tropics toward higher latitudes, except for JSBACH and LPJ‐GUESS, which simulated high values in tropical regions. Our observationally based model showed lower BPE values in some boreal regions, consistent with the predominance of EcM vegetation in temperate and boreal forests. This pattern is consistent with our findings that EcM‐associated ecosystems tend to have low BPE. Empirical BPE estimates ranged from 0.01 to 0.78 and overlapped substantially with TRENDY estimates, which ranged from 0.17 to 0.89 (Figure S6).

## 4 Discussion

Variation in BPE reflects the combined effects of stand development, seasonal climate, soil conditions and biological strategy, rather than a single dominant environmental control. Distinguishing growing‐season from cold‐season temperature effects provides a potential resolution of previously conflicting temperature responses, while variation among forest and mycorrhizal types indicates differences in the carbon costs of tissue maintenance and nutrient acquisition.

The spatial distribution of BPE observations is biased toward Europe and North America, reflecting the availability of long‐term forest data. This imbalance may limit the representativeness of the fitted relationships, particularly in under‐sampled tropical regions. The remaining unexplained variation probably reflects variables not fully captured at the scale of analysis, including ecosystem management (Campioli et al. 2015), as well as substantial unquantified measurement uncertainties (Collalti, Ibrom, et al. 2020).

Forest management was not significantly associated with BPE in our analysis, which may reflect the limited characterization of management type, intensity and duration in the available data. Previous studies have shown higher BPE in managed than in natural forests under comparable environmental conditions (Campioli et al. 2015; Collalti et al. 2018; Collalti and Prentice 2019; Kunert et al. 2019). This may reflect indirect effects of management on site fertility and belowground dynamics (Vicca et al. 2012), as well as reductions in effective stand age and competition for light, water and nutrients (Craine and Dybzinski 2013). Increased nutrient availability under management can also reduce plant dependence on symbiotic associations (Jiang et al. 2025; Treseder 2004), thereby decreasing carbon allocation to belowground partners (Shan et al. 2001) and favoring greater carbon investment in BP, particularly aboveground where light is limiting.

### 4.1 Biological Strategy and Stand Development

BPE did not differ systematically among natural ecosystems with contrasting fertility, averaging 0.46 ± 0.01 across nutrient‐poor tundra and boreal peatlands, nutrient‐rich temperate marshes, and moderately fertile forests and grasslands (Campioli et al. 2015). However, existing evidence indicates systematic differences among vegetation types. Non‐forest vegetation generally has higher BPE than forests, consistent with the smaller standing biomass, more rapid biomass turnover and consequently lower maintenance respiration costs (Collalti, Tjoelker, et al. 2020; Luo et al. 2025). Among forest types, mean BPE has been estimated to be lower in old boreal forests (0.32) than in temperate deciduous forests (0.59) (Collalti and Prentice 2019; De Lucia et al. 2007). This contrast is consistent with our finding of higher BPE in deciduous than evergreen forests, and with independent evidence from high‐frequency CO_2_ flux measurements (Luo et al. 2025). Evergreen species invest in leaves with high leaf mass per area (LMA) (Wang et al. 2023) and amortize these construction costs over multiple growing seasons (Bai et al. 2015; Wang et al. 2023). In contrast, deciduous species invest more in nutrient acquisition to maximize net carbon gain during short growing seasons, while seasonal leaf shedding reduces the maintenance costs associated with long‐lived leaves exposed to freezing or drought (Harrison et al. 2010; Zhang et al. 2024).

Our analysis also identified mycorrhizal strategy as an important biological control on BPE. The lower BPE of EcM and mixed EcM–AM species relative to AM species is consistent with evidence that a significant proportion of plant‐assimilated carbon is allocated to EcM fungi, particularly at higher latitudes (Mäkelä et al. 2022). EcM dominated ecosystems allocate substantial carbon belowground to support fungal biomass, increasing respiratory carbon losses and reducing the fraction of assimilated carbon retained as plant biomass (Clemmensen et al. 2013, 2015; Hobbie 2006; Phillips et al. 2013). They also typically have higher topsoil C:N ratios than AM‐dominated ecosystems, reflecting slower nutrient cycling and greater soil carbon accumulation (Averill et al. 2014; Jiang et al. 2025; Phillips et al. 2013; Read et al. 2004; Terrer et al. 2019). Although EcM fungi can efficiently acquire nitrogen in nutrient‐poor soils, much of this nitrogen is immobilized within fungal biomass, while suppression of saprotrophic decomposition can further reinforce nutrient limitation and increase the carbon costs of nutrient acquisition for host plants (Fernandez and Kennedy 2016; Näsholm et al. 2013). By contrast, AM‐associated plants generally incur lower symbiotic carbon costs and are linked to faster nutrient cycling, with their fungal biomass also responding to nutrient availability (Read and Perez‐Moreno 2003; Treseder and Allen 2002). These differences in carbon allocation and nutrient cycling are consistent with the higher BPE estimated for AM‐associated species.

BPE has previously been shown to decline with increasing forest stand age, and some apparent variation among forest types may reflect differences in their age distributions (Collalti et al. 2018; Doughty et al. 2018). However, the processes underlying this decline remain unresolved. Age‐driven decline in forest BP may result from declining GPP rather than increased respiration (Tang et al. 2014), while declines in both GPP and BP with stand age have also been reported (Collalti et al. 2019; Collalti, Tjoelker, et al. 2020). Age‐related reductions in BP have been attributed to increasing hydraulic constraints on canopy photosynthesis (Hubbard et al. 1999; Ryan and Yoder 1997; Ryan et al. 1997). Taller trees tend to have lower stomatal conductance, photosynthetic rates and leaf‐specific hydraulic conductance, while stomatal closure to avoid xylem cavitation can further reduce GPP (Mencuccini et al. 2005; Ryan et al. 2006). However, our analysis showed a slightly positive overall relationship between stand age and GPP, whereas BPE declined with increasing stand age (Figure 4). This result is more consistent with increasing (predominantly stem) maintenance respiration relative to GPP as stand age as in Collalti, Ibrom, et al. (2020), Collalti, Tjoelker, et al. (2020). Taller trees require greater sapwood mass to support a given leaf area and have longer hydraulic pathways, increasing the metabolic costs of ion transport and cellular maintenance in sapwood and consequently *R*_a_ (Mäkelä and Valentine 2001; Mencuccini et al. 2005; Ryan and Yoder 1997; Sillett et al. 2010). Age‐dependent increases in LMA, associated with thicker and more lignified cell walls and greater leaf density (Poorter et al. 2009), could further increase leaf‐specific respiration costs.

Stand development also alters forest composition through competition for canopy access (Drake et al. 2011), while increasing resource limitation in older stands might decrease BPE. Competition also affects carbon allocation, with light limitation potentially shifting allocation between wood and fine roots while canopy allocation remains relatively stable (Merganičová et al. 2019). The ratio of below‐ground to above‐ground allocation generally declines with stand age, as younger stands allocate a greater proportion of carbon to roots to support water and nutrient uptake under stronger competition (Aakala et al. 2013; Mokany et al. 2006). As stands age, a greater proportion of assimilated carbon is allocated to maintaining existing biomass, non‐structural carbon reserves and chemical defenses rather than to new biomass production (Malhi 2012). These age‐related changes in competition and carbon allocation could further contribute to the observed decline in BPE.

### 4.2 Environmental Controls of BPE

Collalti, Ibrom, et al. (2020) inferred that BPE increases with mean annual temperature, although it declines above a certain temperature. By contrast, analysis of eddy‐covariance flux data has shown that CUE declines with growth temperature, potentially when the increase in *R*_a_ exceeds that in GPP (Luo et al. 2025). Distinguishing between growing‐season and winter temperatures may help reconcile these contrasting responses. Annual BPE depends not only on growing‐season metabolism, but also on respiratory costs associated with entering dormancy before winter and emerging from dormancy in spring (Sperling et al. 2015; Chang et al. 2021). In our analysis, BPE decreased significantly with *T*_g_, but increased significantly with MTCO. Leaf maintenance respiration increases with increasing *T*_g_ (Atkin et al. 2015; Ren et al. 2024), as increased enzyme activity accelerates metabolic rates and increases the energy required for growth, protein transport and turnover, and the maintenance of cellular ion gradients (Di Stefano et al. 2018; Wang, Atkin, et al. 2020). Higher photosynthetic activity can also increase substrate availability for respiration (Wang, Atkin, et al. 2020). The effect of temperature on GPP, however, can be either positive or negative. Rising temperatures have a predominantly positive effect on GPP in northern high latitudes due to cool baseline temperatures and short growing seasons (Piao et al. 2007), but a generally negative effect in subtropical and tropical regions. Consistent with these contrasting regional responses, the P model indicates an overall negative effect of warming on global GPP (Cai and Prentice 2019).

MTCO represents winter thermal conditions. Freezing temperatures can cause sustained photoinhibition, with stronger effects in thinner than in thicker leaves (Cheng et al. 2019). In cold climates, both evergreen and deciduous trees have strategies to avoid frost damage in early spring, leading to a delayed recovery of photosynthetic activity (Luo et al. 2023). Evergreen plants rely on cold acclimation to tolerate low winter temperatures (Öquist and Huner 2003), incurring carbon costs associated with constructing structurally resistant leaves, maintaining frost protection, and repairing winter damage (Barboni et al. 2004). Deciduous trees avoid many foliage‐maintenance costs through leaf shedding, although delayed leaf emergence restricts carbon uptake early in the growing season (Luo et al. 2023). These responses reduce early carbon uptake and may therefore contribute to lower BPE in climates with cold winters. The opposing relationships of BPE with growing‐season and winter temperatures also provide a mechanistic explanation for the previously reported peaked relationship between BPE and mean annual temperature (Piao et al. 2010).

BPE increased with climatic aridity, indicating that forests in drier climates retain a greater fraction of assimilated carbon in biomass, consistent with eddy‐covariance analyzes showing that carbon‐use efficiency declines with increasing precipitation (Luo et al. 2025). Limited water availability directly constrains GPP through stomatal closure and reduced light‐use efficiency (Prentice et al. 2011; Lin et al. 2015; Stocker et al. 2018; Mengoli et al. 2025). However, these reductions in GPP do not necessarily translate into proportional declines in BP and therefore do not inevitably reduce BPE. GPP provides the carbon source for BP, but biomass production can also be constrained by other sink processes (e.g., resource allocation) and therefore respond differently to environmental conditions (Cabon et al. 2022; Kannenberg et al. 2025). Forests with higher turnover and shorter biomass residence times, partly associated with elevated drought‐induced tree mortality, tend to be dominated by fast‐growing species with lower investment in defensive tissues and lower maintenance respiration costs, potentially reducing *R*_a_ and increasing BPE (Malhi et al. 2015). Plant hydraulic strategies may also reduce carbon costs in arid climates by shifting in root: shoot allocation, allowing root systems to access spatially distributed water while limiting investment in and maintenance of below‐ground biomass, thereby reducing respiratory losses (Ding et al. 2025).

Edaphic factors regulate soil fertility and the movement of water and nutrients, thereby influencing resource availability to plants. Soil sand content is inversely related to soil water‐holding capacity and water availability (Zhang et al. 2021). The negative relationship between sand content and BPE may reflect greater carbon allocation to roots for water acquisition in sandy soils, reducing the fraction of assimilated carbon retained as biomass (Wang et al. 2008). Water limitation may also increase respiratory costs through maintaining vascular solute gradients, repairing drought‐induced cellular damage and sustaining futile cycles (Atkin and Macherel 2009; Metcalfe et al. 2010; Wright et al. 2006).

Soil C:N ratio and pH represent different aspects of soil fertility. High soil C:N ratio is an inverse indicator of soil N availability and is negatively related to N mineralization rate (Vicca et al. 2018; Yan et al. 2012). The increase in BPE with decreasing soil C:N ratio is therefore consistent with greater whole‐plant growth when more nitrogen is available (Vicca et al. 2012). Forests with high nutrient availability retain a greater proportion of photosynthate as biomass than nutrient‐poor forests, which allocate relatively more carbon to root‐associated symbionts for nutrient acquisition (Högberg et al. 2003; Vicca et al. 2012). Soil pH affects nutrient availability through interacting chemical, enzymatic, and microbial processes and is also associated with plant physiological traits (Wang, Guo, et al. 2020). Leaf nitrogen per area (*N*_area_) is generally higher at high‐pH sites (Dong et al. 2020; Maire et al. 2015). Although acid soils are often considered to be infertile, low pH has been associated with more open stomata, as inferred from leaf stable carbon isotope measurements, but lower photosynthetic capacity (Chen et al. 2022; Cornwell et al. 2018; Dong et al. 2020; Wang et al. 2017). These contrasting physiological responses may alter below‐ground carbon allocation and help explain the higher BPE estimated at low‐pH sites (Ding et al. 2025).

### 4.3 Outlook

Most TRENDY models showed a similar latitudinal pattern to the data, with BPE increasing with absolute latitude. The lower BPE at low latitudes may partly reflect higher leaf construction costs under greater irradiance, which reduce the proportion of assimilated carbon available for biomass production (Poorter et al. 2006). However, the empirical model deviated from this general pattern in boreal regions, where estimated BPE was relatively low. This reduction is consistent with the predominance of EcM‐associated vegetation and evergreen needleleaf forests at temperate and boreal latitudes, both of which were associated with lower BPE in our analysis (Luo et al. 2025; Mäkelä et al. 2022). The generally higher BPE estimates from TRENDY may reflect differences in the parameterization of plant functional types, among which fast‐growing grasses and crops can achieve higher BPE than forests (Quetin et al. 2020).

The limited agreement with observed BPE may also reflect simplified process representation in TRENDY models. Models lacking age‐dependent growth mechanisms may be limited in simulating the higher biomass accumulation rates characteristic of young forests relative to old‐growth stands (Haverd et al. 2018; Poorter et al. 2016; Wolf et al. 2011). Such limitations are evident in ORCHIDEE, where biomass dynamics are represented using fixed turnover times and allocation fractions, constraining variability in BPE related to differences in stand age, tree size, and environmental stress (Krinner et al. 2005). Processes by which soil properties influence primary production and carbon allocation are also missing from many current models (including the P model). Our analysis therefore points to the importance of representing stand dynamics and soil effects when modeling plant growth.

Our hypothesis testing approach showed quantitative relationships that extend previous observational findings and indicate that BPE is jointly regulated by seasonal climate, soil conditions, stand development, and biological strategy. In particular, the contrasting relationships with growing‐season and winter temperatures help reconcile previously conflicting temperature responses, while differences among forest and mycorrhizal types indicate variation in the carbon costs of tissue maintenance and nutrient acquisition.

Our study relies on a relatively small data set, and there are likely large uncertainties associated with GPP and BP measurements. The lower explanatory power under site‐grouped cross‐validation indicates reduced predictive performance across sites, which may partly reflect the regional sampling imbalance. However, the identified relationships are supported by independent flux observations and regional studies. The sensitivity analysis showed that the broad spatial pattern of BP was partly consistent between the P‐model and TRENDY GPP products, although the overall magnitude and regional distribution were sensitive to the GPP product. Nonetheless, we provide a straightforward explanation for the previously ambiguous sensitivity of BPE to mean annual temperature, and a new empirical benchmark for modeling BP. Our findings emphasize the importance of representing both abiotic and biotic controls on BPE across ecosystems and plant types, including soil and climate controls on biomass allocation. The results further highlight the need to model respiration as an integrated component of whole‐plant function, rather than as either a fixed fraction of GPP or a passive response to enzyme kinetics.

## Author Contributions

**Ruijie Ding:** writing – review and editing, writing – original draft, conceptualization, methodology, formal analysis, software, visualization, data curation, investigation, validation, project administration. **Rodolfo L. B. Nóbrega:** writing – review and editing. **Alessio Collalti:** writing – review and editing, resources, conceptualization. **Sandy P. Harrison:** writing – review and editing, conceptualization. **Iain Colin Prentice:** conceptualization, methodology, funding acquisition, supervision, writing – review and editing.

## Funding

This work was supported by the Schmidt Sciences.

## Conflicts of Interest

The authors declare no conflicts of interest.

## Supporting information


**Table S1:** Summary for coefficients of variables regressed against logit‐transformed biomass production efficiency (BPE) using stepwise selection and LASSO regression.
**Table S2:** In‐sample and cross‐validated performance of the OLS model of logit‐transformed biomass production efficiency.
**Table S3:** Summary statistics for the regression of logit‐transformed biomass production efficiency (BPE) on predictor variables in evergreen species.
**Table S4:** Summary statistics for the regression of logit‐transformed biomass production efficiency (BPE) on predictor variables in deciduous species.
**Table S5:** Summary statistics for linear mixed‐effects model of logit‐transformed biomass production efficiency (BPE) on predictor variables in evergreen and deciduous species.
**Figure S1:** Distributions of environmental variables in the dataset.
**Figure S2:** Observed and cross‐validated predictions of logit‐transformed biomass production efficiency.
**Figure S3:** Pearson correlation coefficients and Variance Inflation Factors (VIF) for different predictors.
**Figure S4:** Sensitivity of global forest biomass production to the choice of GPP product.
**Figure S5:** Statistics for the comparison of global simulations (from our study and eight TRENDY models) interpolated to measurement sites.
**Figure S6:** The global distribution of biomass production efficiency (BPE) estimated by the multiple linear regression model and eight Dynamic Global Vegetation Models (DGVMs) in the TRENDY project, with their latitudinal variations.

## Data Availability

The observed BPE data and the model simulations of this study are available online and can be accessed at https://doi.org/10.5281/zenodo.20272796. The observed and climate values are available online and can be accessed at CHELSA V2.1 (https://chelsa‐climate.org/). Soil data are from GSDE (http://globalchange.bnu.edu.cn/research/soilw), SoilGrid2.0 (https://soilgrids.org/) and WISE30sec (https://www.isric.org/explore/wise‐databases). Soil plant‐available phosphorus (P) was obtained from the Global Database of Soil Plant Available Phosphorus (https://doi.org/10.6084/m9.figshare.1424185). Mycorrhizal type data are from FungalRoot database (https://doi.org/10.1111/nph.16569) and the global mycorrhizal plant distribution map (https://doi.org/10.1038/s41467‐019‐13019‐2). Global forest management data can be accessed at https://zenodo.org/records/5849150. The TRENDY v8 DGVM simulations can be requested on the website (https://blogs.exeter.ac.uk/trendy). *Code availability*: The reproducible R Scripts used to conduct all data manipulations and analyzes are available at: https://doi.org/10.5281/zenodo.20272796.
