# Supplementary material for: Resolving the Multiple Controls of Biomass Production Efficiency in Woody Plants
Source: Glob Chang Biol. 2026 Nov 4;32(11):e71173. doi: 10.1111/gcb.71173 (PMC13732807; doi:10.1111/gcb.71173)
Supplement: Supplementary file 1 [file GCB-32-e71173-s001.docx]

**Supplementary Materials**

**Table S1.** **Summary for coefficients of variables regressed against logit-transformed biomass production efficiency (BPE) using stepwise selection and LASSO regression.** Predictors are growing-season mean temperature (*T_g_*, ˚C), mean temperature of the coldest month (MTCO, ˚C), photosynthetic photon flux density (PPFD, mol m^−2^ d^−1^), natural log-transformed vapor pressure deficit (ln VPD, Pa), natural log-transformed aridity index (ln AI, unitless), natural log-transformed stand age (ln Age, year), natural log-transformed soil C:N ratio (ln C:N, unitless), soil pH (pH, unitless), soil sand content (Sand, % of weight), soil clay content (Clay, % of weight), bulk density (Bd, g cm^-3^) and natural log-transformed available phosphorus (ln P, mg kg^−1^), forest type (deciduous, evergreen) and mycorrhizal type (AM, arbuscular mycorrhizal; EcM, ectomycorrhizal; EcM-AM, mixed colonization by the two types of mycorrhizal fungi).

| Predictor variable | Coefficient  (Stepwise selection) | *p-*value | Coefficient  (Lasso regression) | *p-*value |
| --- | --- | --- | --- | --- |
| Intercept | 4.758 | < 0.001 | 5.213 | < 0.001 |
| *T_g_* (˚C) | $-$0.056 | < 0.001 | / | / |
| MTCO (˚C) | 0.009 | 0.044 | 0.007 | 0.088 |
| ln Age (year) | $-$0.200 | < 0.001 | $-$0.167 | < 0.001 |
| ln C:N (unitless) | $-$0.687 | 0.002 | $-$0.405 | 0.021 |
| pH (unitless) | $-$0.151 | < 0.001 | / | / |
| Sand (% of weight) | $-$0.008 | < 0.001 | $-$0.008 | < 0.001 |
| Bd (g cm^-3^) | $-$0.404 | 0.140 | / | / |
| ln P (mg kg^−1^) | 0.076 | 0.063 | 0.109 | 0.002 |
| ln AI (unitless) | 0.120 | 0.014 | / | / |
| ln VPD (Pa) | / | / | $-$0.590 | < 0.001 |
| Forest_type  evergreen | $-$0.132 | 0.163 | $-$0.133 | 0.099 |
| Mycorrhizal_type  EcM | $-$0.327 | 0.022 | $/$ | $/$ |
| Mycorrhizal_type  EcM-AM | $-$0.328 | 0.100 | $/$ | / |

**Table S2. In-sample and cross-validated performance of the OLS model of logit-transformed biomass production efficiency.** Cross-validated performance was evaluated using 100 repetitions of site-grouped five-fold cross-validation. Sites were defined from unique combinations of latitude and longitude, and all observations from the same site were retained in the same fold. Cross-validated mean and cross-validated SD represent the mean and standard deviation, respectively, of each performance metric across the 100 cross-validation repetitions. The 2.5th and 97.5th percentiles describe the distribution of each metric across the repetitions. RMSE and MAE are expressed on the logit-transformed BPE scale.

| Metric | Full-data estimate | Cross-validated mean | Cross-validated SD | 2.5th percentile | 97.5th percentile |
| --- | --- | --- | --- | --- | --- |
| R^2^ | 0.283 | 0.163 | 0.031 | 0.103 | 0.214 |
| Adjusted R^2^ | 0.261 | / | / | / | / |
| RMSE | 0.565 | 0.610 | 0.011 | 0.591 | 0.632 |
| MAE | 0.420 | 0.461 | 0.010 | 0.444 | 0.480 |

**Table S3.** **Summary statistics for the regression of logit-transformed biomass production efficiency (BPE) on predictor variables in evergreen species.** Predictors are growing-season mean temperature (*T_g_*, ˚C), mean temperature of the coldest month (MTCO, ˚C) , natural log-transformed stand age (ln Age, year), natural log-transformed soil C:N ratio (ln C:N, unitless), soil pH (pH, unitless), soil sand content (Sand, % of weight), natural log-transformed aridity index (ln AI, unitless) and mycorrhizal type (AM, arbuscular mycorrhizal; EcM, ectomycorrhizal; EcM-AM, mixed colonization by the two types of mycorrhizal fungi).

| Predictor variable | Coefficient | SE | *t*-value | *p-*value | *R^2^* | df |
| --- | --- | --- | --- | --- | --- | --- |
| Intercept | 4.421 | 0.975 | 4.533 | < 0.001 |  |  |
| *T_g_* (˚C) | $-$0.073 | 0.017 | $-$4.419 | < 0.001 |  |  |
| MTCO (˚C) | 0.023 | 0.006 | 3.969 | < 0.001 |  |  |
| ln Age (year) | $-$0.120 | 0.043 | $-$2.751 | 0.006 |  |  |
| ln C:N (unitless) | $-$0.497 | 0.232 | $-$2.138 | 0.033 | 0.305 | 207 |
| pH (unitless) | $-$0.383 | 0.062 | $-$6.136 | < 0.001 |  |  |
| Sand (% of weight) | $-$0.009 | 0.003 | $-$3.535 | < 0.001 |  |  |
| ln AI | 0.424 | 0.098 | 4.333 | < 0.001 |  |  |
| Mycorrhizal type EcM | 0.427 | 0.404 | 1.057 | 0.292 |  |  |
| Mycorrhizal type EcM-AM | 0.494 | 0.456 | 1.082 | 0.281 |  |  |

**Table S4.** **Summary statistics for the regression of logit-transformed biomass production efficiency (BPE) on predictor variables in deciduous species.** Environmental factors are growing-season mean temperature (*T_g_*, ˚C), mean temperature of the coldest month (MTCO, ˚C), natural log-transformed stand age (ln Age, year), natural log-transformed soil C:N ratio (ln C:N, unitless), soil pH (pH, unitless) and soil sand content (Sand, % of weight) and natural log-transformed aridity index (ln AI, unitless) and mycorrhizal type (AM, arbuscular mycorrhizal; EcM, ectomycorrhizal; EcM-AM, mixed colonization by the two types of mycorrhizal fungi).

| Predictor variable | Coefficient | SE | *t*-value | *p-*value | *R^2^* | df |
| --- | --- | --- | --- | --- | --- | --- |
| Intercept | 3.138 | 1.080 | 2.906 | 0.004 |  |  |
| *T_g_* (˚C) | $-$0.033 | 0.019 | $-$1.758 | 0.082 |  |  |
| MTCO (˚C) | 0.004 | 0.009 | 0.434 | 0.665 |  |  |
| ln Age (year) | $-$0.250 | 0.068 | $-$3.662 | < 0.001 |  |  |
| ln C:N (unitless) | $-$0.825 | 0.384 | $-$2.149 | 0.033 | 0.376 | 105 |
| pH (unitless) | 0.089 | 0.065 | 1.381 | 0.170 |  |  |
| Sand (% of weight) | $-$0.001 | 0.003 | $-$0.393 | 0.695 |  |  |
| ln AI | 0.272 | 0.169 | $-$1.607 | 0.111 |  |  |
| Mycorrhizal type EcM | $-$0.333 | 0.137 | $-$2.414 | 0.018 |  |  |
| Mycorrhizal type EcM-AM | $-$0.611 | 0.252 | $-$2.423 | 0.017 |  |  |

**Table S5.** **Summary statistics for linear mixed-effects model of logit-transformed biomass production efficiency (BPE) on predictor variables in evergreen and deciduous species.** The model includes site as a random intercept. Predictor variables are growing-season mean temperature (*T_g_*, ˚C), mean temperature of the coldest month (MTCO, ˚C), natural log-transformed stand age (ln Age, year), natural log-transformed soil C:N ratio (ln C:N, unitless), soil pH (pH, unitless) and soil sand content (Sand, % of weight) and natural log-transformed aridity index (ln AI, unitless). Forest type (evergreen, deciduous) and mycorrhizal type (AM, arbuscular mycorrhizal; EcM, ectomycorrhizal; EcM-AM, mixed ectomycorrhizal and arbuscular mycorrhizal colonization) were included as factor.

| Predictor variable | Coefficient | SE | *t*-value |
| --- | --- | --- | --- |
| Intercept | 4.267 | 0.876 | 4.870 |
| *T_g_* (˚C) | $-$0.049 | 0.017 | $-$2.928 |
| MTCO (˚C) | 0.008 | 0.006 | 1.317 |
| ln age (year) | $-$0.175 | 0.040 | $-$4.347 |
| ln CN (unitless) | $-$0.620 | 0.256 | $-$2.423 |
| Sand (% of weight) | $-$0.009 | 0.003 | $-$3.105 |
| pH (unitless) | $-$0.186 | 0.065 | $-$2.880 |
| ln AI | 0.172 | 0.110 | 1.560 |
| Forest type:  evergreen | $-$0.261 | 0.103 | $-$2.531 |
| Mycorrhizal_type:EcM | $-$0.125 | 0.168 | $-$0.743 |
| Mycorrhizal_type:EcM-AM | $-$0.213 | 0.233 | $-$0.915 |

**Fig. S1** **Distributions of environmental variables in the dataset.** Environmental factors are a: growing-season mean temperature (*T_g_*, ˚C), b: mean temperature of the coldest month (MTCO, ˚C), c: photosynthetic photon flux density (PPFD, mol m^−2^ d^−1^), d: vapor pressure deficit (VPD, Pa), e: aridity index (AI, unitless), f: stand age (Age, year), g: soil C:N ratio (CN, fraction), h: soil pH (pH, unitless), i: soil sand content (Sand, % of weight), j: soil clay content (Clay, % of weight), k: bulk density (Bd, g cm^−3^), l: available phosphorus (ln P, mg kg^−1^).

**Fig. S2 Observed and cross-validated predictions of logit-transformed biomass production efficiency.** Predictions were obtained from 100 repetitions of site-grouped five-fold cross-validation. Each point represents an observation, with predicted values averaged across the 100 occasions on which its site was withheld from model fitting. The dashed line represents the 1:1 relationship.

**Fig. S3** **Pearson correlation coefficients and Variance Inflation Factors (VIF) for different predictors.** a: The coefficients are shown by numerical values and the gradient of colors for different environmental variables. b: VIFs for the model with predictors in the multiple linear regression model. The variables are growing-season mean temperature (*T_g_*, ˚C), mean temperature of the coldest month (MTCO, ˚C), natural log-transformed stand age (ln Age, year), natural log-transformed soil C:N ratio (ln CN), soil pH (pH, unitless), soil sand content (Sand, % of weight), natural log-transformed aridity index (ln AI, unitless), forest type and mycorrhizal type

**Fig. S4 Sensitivity of global forest biomass production to the choice of GPP product.** Biomass production was calculated using the same empirically modelled BPE combined with (a) P-model GPP and (b) ensemble-mean GPP from eight TRENDY models. Panel (c) shows the normalized difference, calculated as 100 × (BP_P_−BP_TRENDY_)/(BP_P_+BP_TRENDY_). Positive values indicate higher BP estimates based on P-model GPP. BP is expressed in g C m⁻² yr⁻¹.

**Fig. S5** Statistics for the comparison of global simulations (from our study and eight TRENDY models) interpolated to measurement sites. “Empirical” refers to the multiple linear regression model. Dashed lines represent the 1:1 lines.

**Fig. S6** The global distribution of biomass production efficiency (BPE) estimated by the multiple linear regression model and eight Dynamic Global Vegetation Models (DGVMs) in the TRENDY project, with their latitudinal variations.
